# Optimization of the biotechnological production of a novel class of anti-MRSA antibiotics from *Chitinophaga sancti*

**DOI:** 10.1186/s12934-017-0756-z

**Published:** 2017-08-17

**Authors:** Amelie Beckmann, Stephan Hüttel, Viktoria Schmitt, Rolf Müller, Marc Stadler

**Affiliations:** 1 Department of Microbial Drugs, Helmholtz Centre for Infection Research, Brunswick, Germany; 2 Helmholtz-Institute for Pharmaceutical Research Saarland, Helmholtz Centre for Infection Research and Dept. Pharmaceutical Biotechnology of Saarland University, Saarbrücken, Germany; 3 German Centre for Infection Research (DZIF), Partner Site Hannover-Braunschweig, Brunswick, Germany

**Keywords:** Antibacterials, Antibiotic resistance, Bioprocess optimization, MRSA, Secondary metabolites

## Abstract

**Background:**

Recently, the discovery of the elansolids, a group of macrolides, was reported. The molecules show activity against methicillin-resistant *Staphylococcus aureus* as well as other gram-positive organisms. This fact renders those substances a promising starting point for future chemical development. The active atropisomers A1/A2 are formed by macrolactonization of the biosynthesis product A3 but are prone to ring opening and subsequent formation of several unwanted side products. Recently it could be shown that addition of different nucleophiles to culture extracts of *Chitinophaga sancti* enable the formation of new stable elansolid derivatives. Furthermore, addition of such a nucleophile directly into the culture led exclusively to formation of a single active elansolid derivative. Due to low product yields, methods for production of gram amounts of these molecules have to be established to enable further development of this promising compound class.

**Results:**

Production of elansolid A2 by *C. sancti* was enabled using a synthetic medium with sucrose as carbon source to a final concentration of 18.9 mg L^−1^. A fed-batch fermentation was ensued that resulted in an elansolid A2 concentration of 55.3 mg L^−1^. When using glucose as carbon source in a fed-batch fermentation only 34.4 mg L^−1^ elansolid A2 but 223.1 mg L^−1^ elansolid C1 were produced. This finding was not unexpected since elansolids A1/A2 and A3 have been reported to easily react with nucleophiles like anthranilic acid, a precursor of tryptophan biosynthesis. Due to the fact that nucleophiles can be incorporated in vivo, a fed-batch cultivation under identical conditions, with addition of anthranilic acid was carried out and lead to almost exclusive formation of elansolid C1 (257.5 mg L^−1^).

**Conclusion:**

Reproducible elansolid A2 and C1 production is feasible in different synthetic media at relatively high concentrations that will allow further investigation and semi-synthetic optimization. The feeding of anthranilic acid enables the exclusive production of the stable elansolid derivative C1, which reduces product loss by unspecific reactions and eases downstream processing. This derivative shows activity in the same range as the elansolids A1/A2. Hence, the method can possibly serve as a model-process for incorporation of other nucleophiles and biotechnological production of specifically designed molecules.

## Background

Methicillin-resistant *Staphylococcus aureus* (MRSA) is at present the most commonly identified antibiotic-resistant pathogen in many parts of the world with a prevalence between 25 and 50% in most parts of America, Australia and southern Europe [1, 2]. In 2005, 19,000 deaths associated with MRSA strains were reported in the USA [3]. Although infection rates are decreasing, MRSA infections were estimated to affect more than 150,000 patients in the EU alone in 2010 [4]. Until recently, vancomycin and daptomycin have been the only effective treatment for methicillin-resistant pathogens [5]. But in 1997, the first vancomycin-intermediate-resistant *S. aureus* strains were isolated, followed by reports of vancomycin-resistant ones in 2003 [6, 7]. Cases of daptomycin-resistant MRSA have also been described [8, 9]. Consequently, the need for new antibiotics for the treatment of these multi-resistant bacteria arises. In a global priority list released by the WHO in 2017, development of antibiotics against MRSA is given the second highest priority [10]. To tackle this threat, efforts are undertaken to find new substances which can be developed as antibacterials. Our onset for discovery of novel molecules which show the desired biological activity is the investigation of culture-extracts from soil microorganisms which lead recently to the discovery of cystobactamids from myxobacteria [11]. Moreover, the current medical need necessitates re-investigation of known substances like the chelocardins, where molecular engineering lead to novel chelocardin derivatives with a broadened gram-negative activity spectrum [12]. Beside those gram-negative acting compounds other molecules like the disciformycins have been discovered in our laboratories, which show only a narrow-spectrum activity against gram-positive bacteria [13] but examples like the fourth generation cephalosporins have shown that the target spectrum can be expanded from solely gram-positive to a broad spectrum of gram-positive and gram-negative bacteria through medicinal chemistry approaches [14–16]. This underlines the importance of the investigation of any novel or under-investigated antibacterial activity since it harbor’s the possibility to address novel antibacterial targets or to overcome resistance problems associated with known antibacterial-targets.

**Scheme 1:**
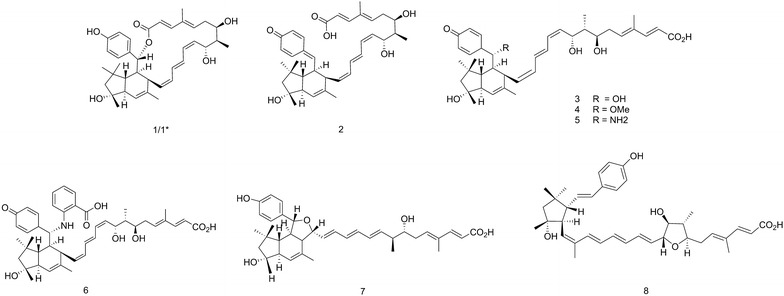
Chemical structures of elansolids. 1/1* elansolid A1/A2, *2* elansolid A3, *3* elansolid B1, *4* elansolid B2, *5* elansolid B3, *6* elansolid C1, *7* elansolid D1, *8* elansolid D2

**Fig. 1:**
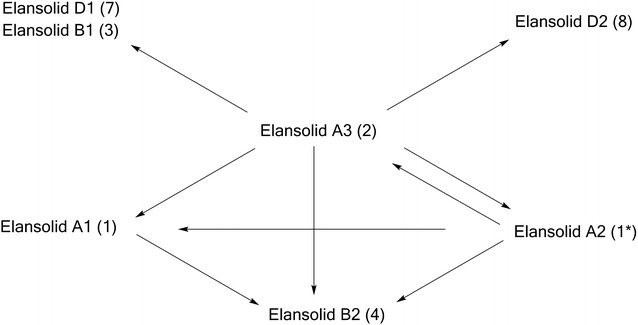
Interconversion of elansolids (adapted from [20])

**Fig. 2:**
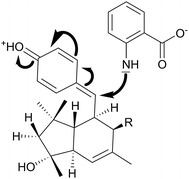
Reaction mechanism for the formation of elansolid C1 (6) by a Michael-type nucleophilic attack of anthranilic acid on the *p*-quinone moiety of elansolid A3 (2). Anthranilic acid can be substituted by other nucleophiles to yield the corresponding derivative

During the search for novel antibiotics, Steinmetz et al. found a group of new macrolides, for which they proposed the trivial names, elansolids (Scheme 1), in the culture extracts of the gram-negative soil-dwelling bacterium *Chitinophaga sancti* [17, 18]. The genus *Chitinophaga* comprises a variety of strains that are promising sources of interesting novel secondary metabolites. Just recently the discovery of the first antifungal lantibiotics, called pinensins, from *Chitinophaga pinensis* has been reported [19]. Some of the elansolids (Fig. 1) exhibited moderate to strong activity against several gram-positive bacterial pathogens including MRSA. Extensive chemical and biological studies revealed that elansolids A1 and A2 (1/1*) occur as two distinct atropisomers. Both elansolids exhibit potent activity against gram-positive bacteria, elansolid A2 being generally more active than elansolid A1. The MIC (minimal inhibiting concentration) of elansolid A2 against methicillin-resistant *S. aureus* strain MRS3 is in the range of 2 µg mL^−1^ [17]. Further studies revealed that the final product of the biosynthetic pathway is deoxyelansolid A3 (2) which undergoes lactonization by an intramolecular Michael addition to yield elansolid A1/A2 [18, 20]. Water, methanol or ammonia can also serve as nucleophiles that attack the quinone methide moiety of elansolid A3 to generate elansolids B1–B3 (3–5) and D1–D2 (7–8) [20]. This reaction can be exploited in two different ways: By adding different precursors as nucleophiles to the crude extracts of a *C. sancti* culture to create new elansolid derivatives in a precursor-directed synthesis or by feeding precursors such as anthranilic acid directly to the culture medium for a directed biosynthesis (Fig. 2). Interestingly, the bioactivities of these newly formed derivatives were roughly in the same range as that of elansolid A2 [21]. These results are promising as they possibly allow the creation of precisely tailored derivatives with increased efficacy, once mode of action studies have revealed the target of the elansolids.

To turn this highly interesting class of novel antibiotics into a lead for pharmaceutical development, it will be necessary to improve both their stability and potency. The compound class is amenable to various structural modifications by means of medicinal chemistry. However, for this purpose, the yields from biotechnological production need to be improved, in order to provide access to the starting material in multi gram scale.

Elansolid A3, which has hitherto been used as starting material for semi-synthesis approaches is highly unstable. Our rationale to direct the biotechnological production in order to allow for easy access of the starting material for a subsequent medicinal chemistry program is that it should be more feasible to produce elansolid A2. The latter compound can be converted back to elansolid A3 under mild conditions [17, 20]. For this reason, the biotechnological production of elansolid A2 with *C. sancti* was investigated in this study. One drawback of the biotechnological production has so far been the formation of several side products—especially in complex media—due to the reactivity of the elansolids A1–A3, which significantly lowered the yield of elansolid A2 in such a process. The reduced yield in undefined media arises from unspecific side reactions with either nucleophilic media components or intermediates, which are released by the producer itself due to cell lysis occuring in complex media when toxic metabolites accumulate. This might be circumvented by reducing formation of toxic metabolites and increasing the viability of the producer with the usage of a defined media and by concurrently directing the product formation towards a stable derivative through feeding of the corresponding precursor. The feasibility of this precursor-directed approach was explored in this study by feeding anthranilic acid, the precursor of elansolid C1 (6) which was shown to be incorporated in vivo and could thereby serve as a model system for direct incorporation of selected building blocks during the production process.

## Methods

### Media and supplements

For seed-cultures, medium (10.0 g L^−1^ soy peptone (Cargill), 1.0 g L^−1^ CaCl_2_·2 H_2_O, 1.0 g L^−1^ MgSO_4_·7 H_2_O, 8.0 mg L^−1^ Na–Fe-EDTA, 11.9 g L^−1^ HEPES; pH 7.4) was used to which maltose monohydrate was added after autoclaving to a final concentration of 10.5 g L^−1^. Bioreactor cultivations were conducted with a production medium (1.99 g L^−1^ (NH_4_)_2_SO_4_, 700 mg L^−1^ KH_2_PO_4_, 200 mg L^−1^ MgSO_4_·7 H_2_O, 8.0 mg L^−1^ Na–Fe-EDTA, 5.0 mg L^−1^ MnSO_4_·H_2_O, 1.0 mg L^−1^ ZnCl_2_; pH 7.0) to which the carbon sources were added after autoclaving giving the following final concentrations: 10.0 g L^−1^ sucrose; 10.1 g L^−1^ glucose monohydrate. To enable elansolid C1 production an anthranilic acid solution in methanol was prepared through sterile filtration and added to the bioreactor before inoculation to give a final concentration of 100 µg L^−1^. To capture the metabolites amberlite XAD16 resin (Sigma) was added to all production media to a concentration of 20 g L^−1^. Feed solutions contained 440 g L^−1^ glucose monohydrate and 146.7 g L^−1^ (NH_4_)_2_SO_4_ that were autoclaved separately and afterwards merged to add up to 500 mL.

### Shake flask cultivations

250 mL shake flasks containing 100 mL pre-culture medium were inoculated with 1.8 mL cryo stock of *Chitinophaga sancti* Fx7914 and incubated at 25 °C and 160 rpm for 24 h. From this seed-culture, bioreactors were inoculated to a concentration of 10 g L^−1^.

### Bioreactor cultivations

All bioreactor cultivations were performed with the parallel cultivation system DASGIP (Eppendorf) and DASware control 5 for process control. The vessels had a total volume of 2.0 L. The system was equipped with optical DO electrodes (Hamilton) and pH electrodes (Hamilton). The off-gas analyzer consisted of zirconium dioxide sensors for O_2_ measurement and infrared sensors for CO_2_ measurement (BlueSens). The DO concentration in the medium was kept at a constant level of 20% by increasing the stirrer speed (200–1200 rpm) and adding pure oxygen to the gas mix if necessary, realized by an internal cascade. The gas flow rate was kept constant at 0.05 vvm. The pH level was kept constant as well by the addition of 50 g L^−1^ H_2_SO_4_ and 50 g L^−1^ KOH. When foaming occurred, Tegosipon anti foam (Evonik) was added with a 1 mL syringe. Batch fermentations were started with a cultivation volume of 1.5 L and lasted 85 h. The fed-batch fermentations were started with a cultivation volume of 1.0 L and lasted 94–190 h. The feed was started after 48 h for the sucrose glucose fermentation and after 24 h for the glucose fermentations with a feeding rate of F = 2.04·e^0.056·t^ mL h^−1^.

### Determination of optical density and substrate concentration

Optical density was determined with a Libra S11 photometer (Biochrom) at λ = 600 nm. Samples were diluted to be in the linear range of the photometer. Sucrose concentrations and corresponding monomeric glucose and fructose were determined with a photometric assay kit from r-biopharm (Art. No. 10716260035). For measuring of glucose concentration, a second method was applied using an Agilent 1260 series HPLC and a Phenomenex REZEX ROA-Organic Acid H+ (8%) column (300 mm × 7.8 mm × 8 µm) at 65 °C with a RID detector and an isocratic gradient of 0.05 mM H_2_SO_4_ for 45 min.

### Adsorber resin extraction and determination of product concentration

After sample taking the amberlite XAD16 was filtered through gauze. The residual resin was weighed and the tenfold volume (w/v) of a 1% acetic acid solution in acetone (v/v) was added to the resin. After 10 min of incubation on a horizontal shaker 1 mL supernatant was centrifuged at 20,000*g* for 10 min. The supernatant was injected into an Agilent 1200 Series HPLC with a Waters Acquity UPLC BEH C18 column (50 mm × 2.1 mm × 1.7 µm) at 40 °C using 0.01% formic acid in water (v/v) and 0.01% formic acid in acetonitrile (v/v) as mobile phase. The gradient went linearly from 5 to 100% acetonitrile in 20 min. The UV signals were detected at *λ* = 220 nm. The product concentrations were determined via a calibration curve using five defined standard solutions.

### Serial dilution assay for determination of antibacterial activity

Minimum inhibitory concentration (MIC) values were determined in standard microbroth dilution assays as described in [22]. In brief, bacterial suspensions of *Escherichia coli* DSM-1116 and TolC-deficient *E. coli* were prepared in Müller-Hinton broth (10^4^–10^5^ cfu mL^−1^). In addition, the efflux-deficient *E. coli* strain was permeabilized with polymyxin B nonapeptide (PMBN) at a sub-inhibitory concentration (3 µg mL^−1^) and used for testing following the same procedure. Given MIC values are the lowest concentrations of antibiotic at which there was no visible growth.

## Results

In previous studies, the activity spectrum of the elansolids was determined to be only gram-positive bacteria, like *S. aureus*. To validate if the molecular target of the elansolids also exists in gram-negative organisms and activity is reduced by reduced membrane diffusion or efflux, a bioactivity assay with the secretion-deficient *E. coli* tolC in combination with the membrane-permeabilizing peptide PMBN was performed revealing that elansolids A2 and C1 inhibit this gram-negative strain with 2 µg mL^−1^ and 8 µg mL^−1^, respectively (Table 1). Following this logic, the elansolids presumably act on the same molecular target in gram-positive and gram-negative bacteria, but cannot penetrate the membrane of gram-negatives [23].

**Table 1:** Minimum inhibitory concentrations (MIC) in µg mL^−1^ of elansolids A2 and C1 against different *E. coli* strains

Strain	Elansolid A2	Elansolid C1
*E. coli* DSM1116	>64	>64
*E. coli* TolC	64	64
*E. coli* TolC + PMBN	2	8

According to Steinmetz et al. [21] it is crucial to use a defined medium without complex media components when producing elansolid A2 because otherwise elansolid C1, a derivative containing anthranilic acid, is produced instead of elansolid A2. A defined medium was developed for which sucrose was found to be the most suitable carbon source for elansolid A2 production during shake flask experiments with an elansolid A2 concentration of around 18 mg L^−1^ (data not shown).

**Fig. 3:**
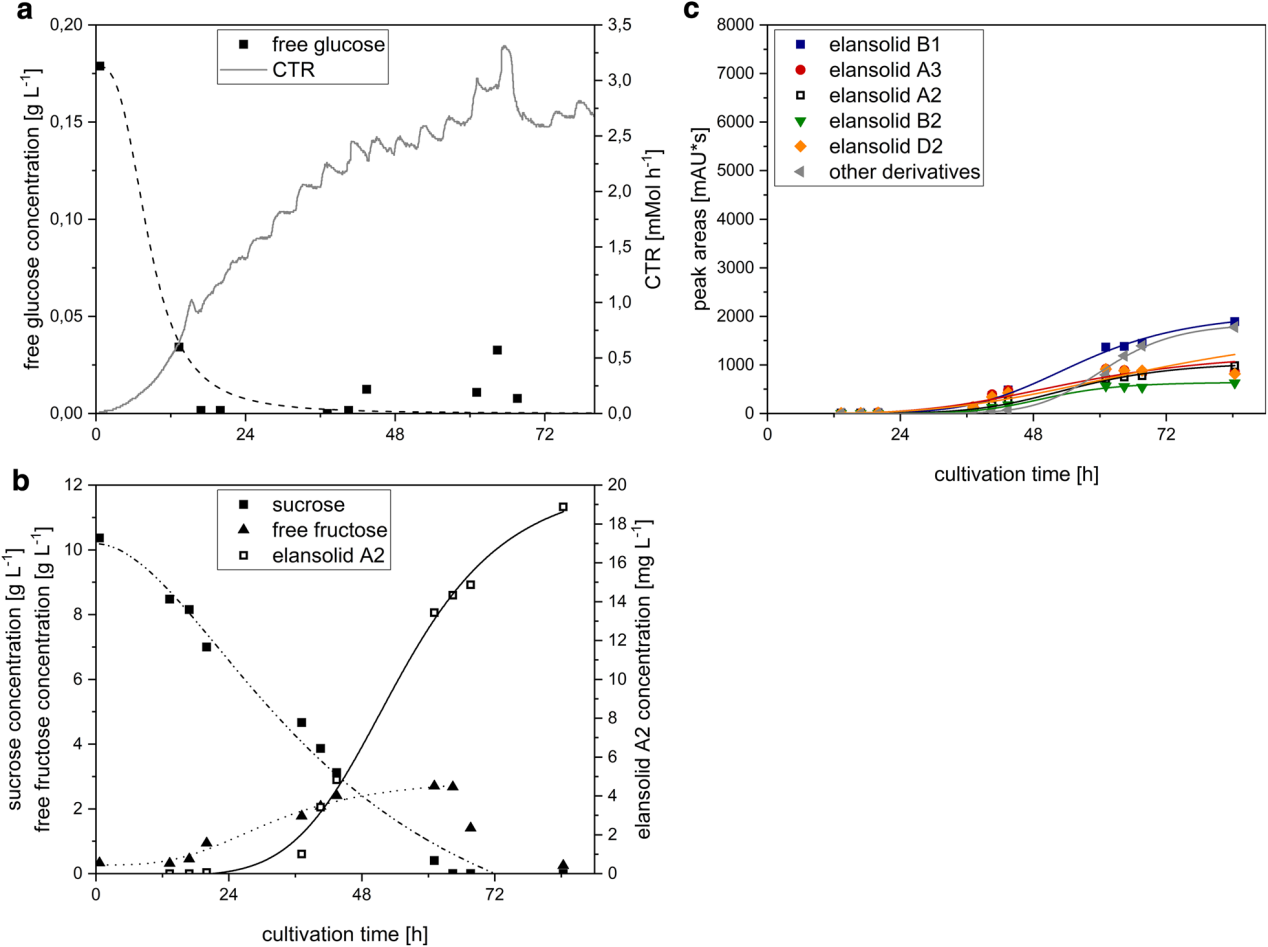
**a** Course of carbon dioxide transfer rate (CTR), free glucose concentration and **b** sucrose, free fructose and elansolid A2 concentrations and **c** peak areas of elansolid derivatives from the HPLC runs throughout the bioreactor batch fermentation of *C. sancti* with sucrose as carbon source

The results were transferred to bioreactor scale, where a batch fermentation in the minimal medium with sucrose as carbon source was conducted (Fig. 3). The disaccharide sucrose was metabolized right from the beginning, but released fructose accumulated in the medium until 61 h after inoculation when the concentration had its highest value with 2.7 g L^−1^. Released glucose could only be detected at the beginning of the fermentation with a concentration of 0.18 g L^−1^ until 13 h (0.03 g L^−1^) after inoculation but vanished within less than 24 h. This indicated that sucrose was probably digested by extracellular invertases and glucose was taken up preferentially by the cells until sucrose was depleted. Afterwards, the remaining fructose was metabolized. After 18 h, a change from exponential to limited growth could be observed, as indicated by the CTR (carbon dioxide transfer rate) (Fig. 3a). This might be due to cleavage of sucrose and supply of glucose as a limiting factor. After 37 h elansolid A2 production could be detected with a sigmoidal increase until the end of the cultivation to a final value of 18.9 mg L^−1^ comparable to the shake flask experiments. As illustrated in Fig. 3c, elansolid A2 is not the only derivative produced. In fact, it accounted for only 14% of the total elansolid yield based on the peak areas of the HPLC runs where the sum of all peak areas is accounted for as 100%. The main product is elansolid B1 (water addition to elansolid A3). Although elansolid A3 is unstable in an aqueous environment it can still be detected, presumably due to slow reaction kinetics or possible protection when bound to the adsorber resin. Besides the published elansolid derivatives there are also several others, which are detectable in minor amounts but together make up a substantial fraction of the overall elansolid yield.

**Fig. 4:**
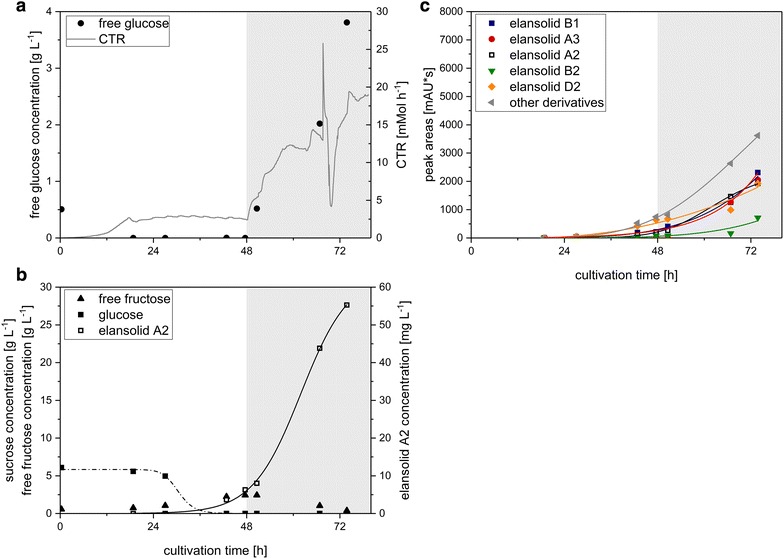
**a** Course of carbon dioxide transfer rate (CTR) and free glucose concentration, **b** sucrose, free fructose and elansolid A2 concentrations, **c** peak areas of elansolid derivatives from the HPLC runs throughout the bioreactor fed-batch fermentation with sucrose in the basal medium and glucose in the feed solution. The *gray areas* represent the duration of feeding

The batch process with sucrose did not lead to an increase in elansolid A2 concentration compared to the shake flask cultivation. Hence, as a next step towards the aim of improved elansolid A2 yield, a fed-batch fermentation was conducted. Sucrose was provided in the medium at the beginning of the fermentation with a concentration of 20 g L^−1^, using the starting conditions of the batch fermentation. Due to preferred utilization of glucose by *C. sancti* in the previous experiment, this sugar was chosen as substrate in the feeding solution. The fermentation was started as a batch cultivation and after 48 h an exponentially increasing glucose feed was started. As seen in Fig. 4, the curve-progression of the illustrated parameters develops similarly to the batch fermentation until the feed was started after 48 h, also exhibiting the transition from exponential to linear growth after 18 h. After starting the feed the CTR rapidly increased from 2.4 to 12.3 mMol h^−1^ after 59 h. The elansolid A2 concentration increased until 74 h to 55.3 mg L^−1^ resulting in a 2.9-fold increase in comparison to the batch fermentation. As in the batch fermentation, several elansolid derivatives were produced of which elansolid A2 made up only 15% of the overall yield (Fig. 4c).

**Fig. 5:**
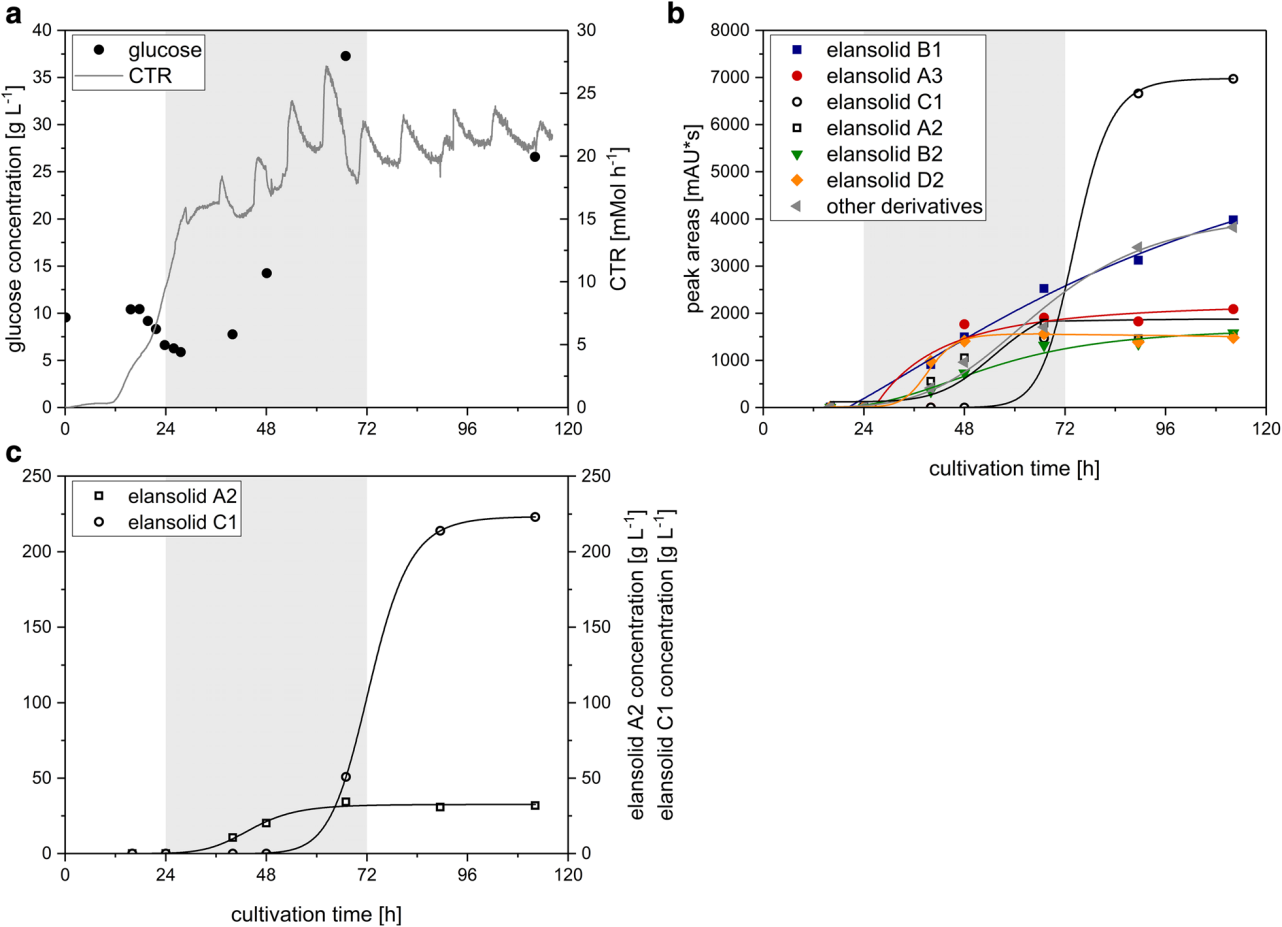
**a** Course of carbon dioxide transfer rate (CTR) and free glucose concentration, **b** sucrose, free fructose and elansolid A2 concentrations, **c** peak areas of elansolid derivatives from the HPLC runs throughout the bioreactor fed-batch fermentation with glucose in the basal medium and glucose in the feed solution. The *gray areas* represent the duration of feeding

Since sucrose as carbon source caused a diauxic growth of *C. sancti,* a second fed-batch fermentation with glucose as sole carbon source in batch and feed-phase was conducted (Fig. 5). The feed was started after 24 h. The CTR increased exponentially in the first 28 h after which a small dent occurred which correlates with the starting point of elansolid A2 production. Following the dent, the CTR increased linearly to 21.0 mMol h^−1^ in average from 48 to 116 h. The oscillation of the CTR is caused by the fact that whenever the culture becomes more acidic, carbon dioxide that was formerly dissolved in the medium is released. When the pH is regulated by adding potassium hydroxide more carbon dioxide can be dissolved again. Therefore, these fluctuations do not represent the true carbon dioxide emission rate (CER), a problem that has been addressed by several publications before [24–26]. The elansolid A2 concentration increased to 34.4 mg L^−1^ after 67 h which was less than in the fermentation with sucrose in the starting medium (end concentration was 31.8 mg L^−1^). This is due to the fact that elansolid C1 was detected in high concentrations from 67 h onwards which was so far only encountered when complex substrates were present in the medium [21]. The final concentration of elansolid C1 was 223.1 mg L^−1^ and accounted for 32% of all derivatives.

**Fig. 6:**
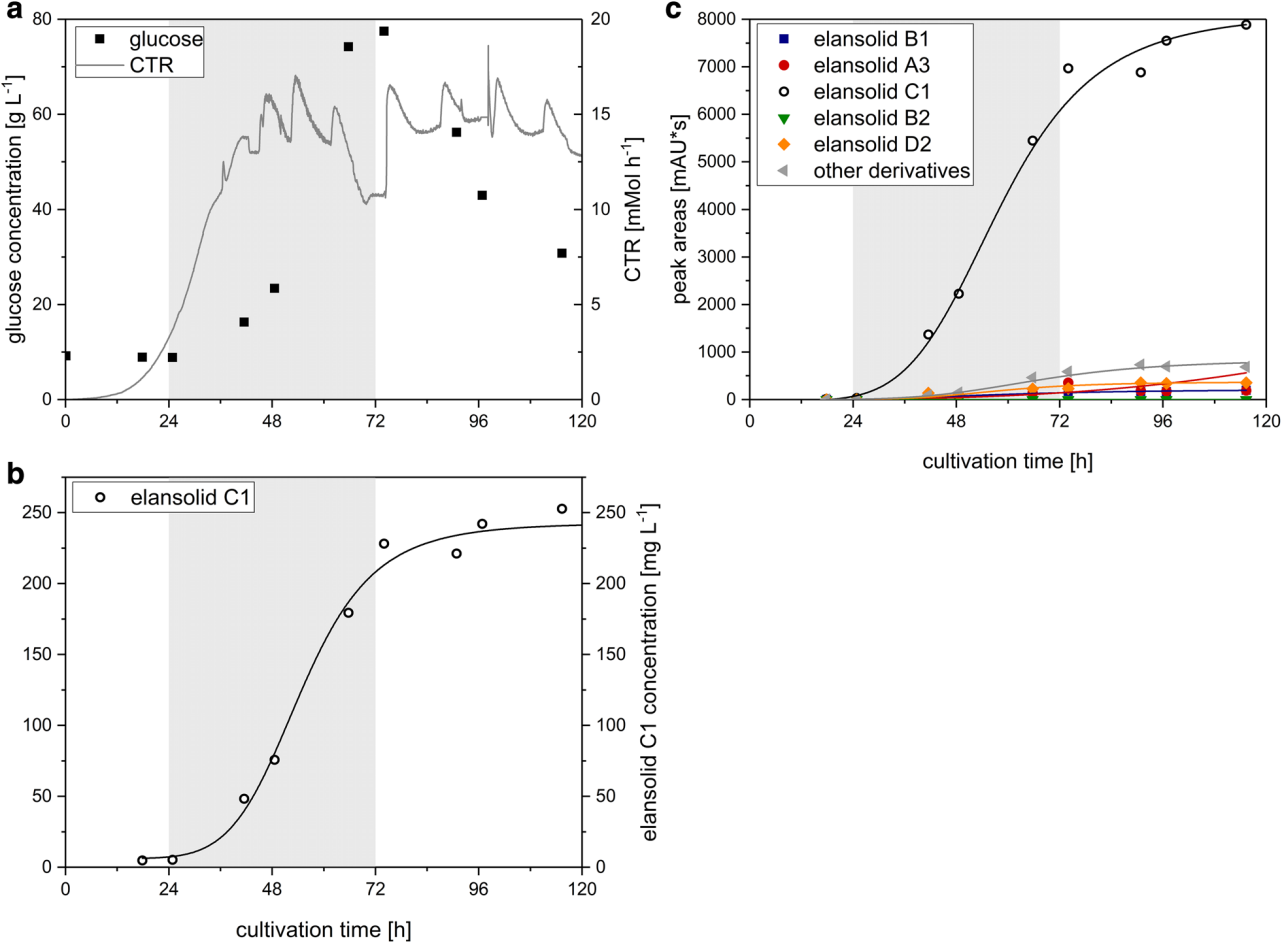
**a** Course of carbon dioxide transfer rate (CTR) and free glucose concentration, **b** sucrose, free fructose and elansolid A2 concentrations, **c** peak areas of elansolid derivatives from the HPLC runs throughout the bioreactor fed-batch fermentation with glucose and anthranilic acid in the basal medium and glucose in the feed solution. The *gray areas* represent the duration of feeding

The accumulation of undesired side products in all cultivations for elansolid A2 production justified the investigation of a process aiming for the exclusive production of a single derivative. As a model system elansolid C1 was chosen since the precursor is readily available and C1 has similar activities as A1/A2 [21]. So far, the directed biosynthesis of elansolid C1 with complex medium yielded product titers with concentrations of around 7.1 mg L^−1^ in batch-fermentations [21]. To show the feasibility of the directed biosynthesis of elansolid C1 in defined medium, a fed-batch fermentation with anthranilic acid supplementation to the medium was conducted. The results are shown in Fig. 6. The respirational activity, represented by the CTR, reached an average of 14.3 mMol h^−1^ from 48 to 120 h and therefore only 68% of the CTR of the fed-batch with glucose as sole carbon source without anthranilic acid (Fig. 5). As a consequence of the reduced metabolic activity, glucose accumulated to a final concentration of 77.5 mg L^−1^. First elansolid C1 was detected after 17 h followed by an exponential increase with a maximum titer of 252.7 mg L^−1^ after 115 h. In this fermentation, the desired derivative elansolid C1 made up the major portion of the total yield with 85% based on the peak areas.

## Discussion

Elansolids are interesting candidates for drug development, but chemical synthesis is to date only available for elansolid B1 [27] and not feasible in multi-gram scale. Therefore, provision of substantial amounts of elansolids to enable further semi-synthetic drug development is only achievable by biotechnological means. The chosen strategy was two-fold: Our first approach aimed towards the production of elansolid A2. This is an ideal starting point for chemical optimization of the molecule where the ring-structure shall be retained whereas our second approach aimed towards the biotechnological production of a linearized derivative by adding a nucleophile (in our case anthranilic acid) to the culture, thereby preventing the accumulation of undesired side products.

In the batch fermentation, we could show that sucrose was cleaved and the monosaccharides were metabolized separately, with glucose as the preferred substrate, yielding 18.9 mg L^−1^ elansolid A2. The cleavage of sucrose might be carried out by extracellular invertases. These findings are well in accordance with publications showing that *Chitinophaga pinensis*, a close relative of *C. sancti*, possesses a variety of carbohydrate-active enzymes (CAZymes) for the degradation of carbohydrates, among them invertase [28–31]. The preferred usage of glucose over other carbon sources has been well observed in different types of organisms under the term carbon catabolite repression [32, 33].

To increase elansolid A2 production, a fed-batch fermentation was applied. Sucrose was chosen as the carbon source in the basal medium whereas glucose was provided in the feed solution as it was shown to enable rapid growth. Hence, the product concentration could be increased almost threefold from 18.8 mg L^−1^ in the batch fermentation to 55.3 mg L^−1^ in the fed-batch fermentation.

The fed-batch fermentation with glucose as sole carbon source resulted in only 31.8 mg L^−1^ elansolid A2 but 223.1 mg L^−1^ elansolid C1. Elansolid C1 is generated when anthranilic acid acts as a nucleophile that attacks the quinone methide ring of elansolid A3 [21]. Since in this process anthranilic acid must be produced by the organism itself presumably as a precursor for tryptophan biosynthesis, accumulation of anthranilic acid and its subsequent side reactions with elansolid A3 could possibly be prevented by feeding tryptophan and thereby exploiting a possible feedback inhibition of the trp-operon [34]. This will be subject of further research for process improvement. Furthermore, the feeding rates need to be adapted to avoid glucose accumulation during the feeding phase. In two of the fed-batch fermentations, glucose accumulated to final concentrations of 37.3 and 77.5 mg L^−1^ and although growth and product formation did not seem to be affected by a potential overflow metabolism, the accumulation of not metabolized substrates reduces process profitability and should therefore be minimized in future experiments.

To prove the concept of precursor-directed biosynthesis of novel elansolid derivatives that harbor several interesting advantages, anthranilic acid was provided in the medium as a precursor in another fermentation. Here, an elansolid C1 concentration of 257.5 mg L^−1^ has been obtained. This yield is equivalent to a 36-fold increase in product concentration compared to 7.1 mg L^−1^ which could be achieved by fermentation so far.

The precursor-directed biosynthesis during fermentation when providing anthranilic acid allowed the formation of the desired product in high amounts whereas formation of other elansolid derivatives occurred only in minor concentrations (85% elansolid C1, Fig. 6c). Accordingly, this procedure should be further investigated as basis for the creation of different novel elansolid derivatives, since the semi-synthetic approach within crude extracts proposed by Steinmetz et al. [21] is accompanied by a considerable loss of product.

The precursor-directed elansolid production is of special interest since although the absolute amount of elansolid A2 was increased considerably comparing absolute product titers of batch fermentation and fed-batch fermentation with sucrose, its share of the overall elansolid yield was similar with 14% in the batch (Fig. 3c) and 15% in the fed-batch process (Fig. 4c). The presence of these side-products increases the workload for purification of elansolid A2. Subsequently, after chromatographic purification of elansolid A2, which is additionally hampered by the stability of the molecule, the compound must be chemically converted into the desired product. This can only be accomplished with considerable losses at this time [20].

In this light, the precursor-directed synthesis of new elansolid derivatives directly during the fermentation might provide a promising tool in the search for novel antibiotics, especially since conversion into the more stable non-cyclic derivative elansolid C1 is accompanied by only a minor loss of activity even when observing gram-negative permeabilized cells. Thus, one focus of medicinal chemistry approaches could be the structure modification towards increased cell wall penetration. Another way to ensure antibiotic uptake is the formation of siderophore drug conjugates [35, 36]. Siderophores are high affinity Fe^3+^ chelators that are used for iron acquisition. The resulting Fe^3+^-siderophore complex is then assimilated through an active transport system by the bacteria. Hence, these siderophores could be coupled to antimicrobial compounds that enter the cell as a ‘Trojan horse’. Nonetheless, additional feeding experiments with different precursors will be conducted to fully prove the feasibility of this concept.
